# Norovirus Gastroenteritis, Carbohydrate Receptors, and Animal Models

**DOI:** 10.1371/journal.ppat.1000983

**Published:** 2010-08-26

**Authors:** Ming Tan, Xi Jiang

**Affiliations:** Division of Infectious Diseases, Cincinnati Children's Hospital Medical Center, University of Cincinnati College of Medicine, Cincinnati, Ohio, United States of America; University of California San Francisco, United States of America

## HBGAs Are an Important Factor in Norovirus Evolution

**Figure 1:**
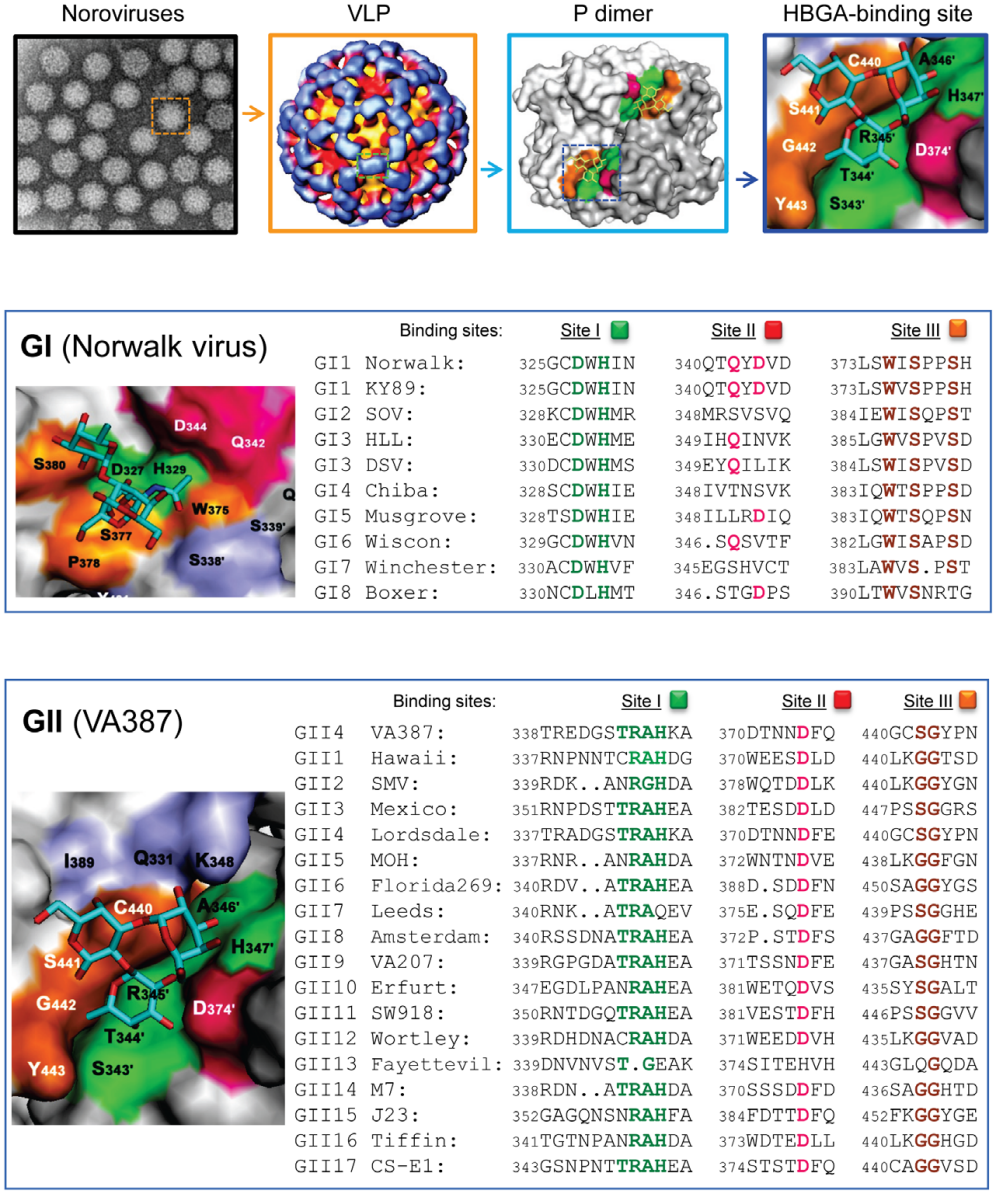
Elucidation of the HBGA-binding pocket and the genetic relatedness of HBGA-binding interfaces among different genotypes of human noroviruses. The top four panels show structures of noroviruses at different levels: (from left to right) an electron microscopy image of noroviruses, a single virus-like particle (VLP), a P dimer with indication of the carbohydrate-binding interface (colored region), and the crystal structure of the HBGA-binding interface. The dashed square in each left panel is enlarged in the right panel. The HBGA is indicated by a ring-shaped trisaccharide. The middle panel shows the crystal structures of the HBGA-binding interface of the prototype Norwalk virus (GI.1, left) and the aligned sequences of the receptor-binding interface of eight GI genotypes (right). The bottom panel shows the crystal structures of the HBGA-binding interface of strain VA387 (GII.4, left) and the aligned sequences of the receptor-binding interface of 17 GII genotypes (right). The HBGA-binding interface can be divided into three sites representing the bottom (green) and walls (orange and red) of the binding pocket. The same color schemes are used in the sequence alignments to highlight the conserved amino acid residues of the three sites. Partially adapted, with permission, from [8].

Noroviruses, an important cause of acute gastroenteritis in humans, have been found to recognize the histo-blood group antigens (HBGAs) as receptors. Different noroviruses revealed different receptor-binding profiles associated with the ABO, secretor, and Lewis HBGA types. Direct evidence of HBGA receptor recognition in viral infection and tropism was obtained from human volunteer challenge studies on the prototype Norwalk virus, in which the infection rates of the volunteers matched well with the HBGA-binding profiles of the challenge virus [1], [2]. Similar evidence was also obtained from investigation of outbreaks of gastroenteritis related to other genotypes of noroviruses [3], [4], although conflicting results also were reported. The HBGA-binding interfaces have been identified in the protruding (P) domain of the viral capsid protein, in which a group of scatted amino acids forms a conformational pocket on the distal surface of the viral capsid that interacts with individual oligosaccharide residues of the HBGA receptors [5]–[7] (Figure 1). These data indicate that the P domain is the primary site of receptor interaction, which plays an essential role in norovirus infection.

The crystal structures of the HBGA-binding interfaces of Norwalk virus (GI.1) and VA387 (GII.4) have been elucidated, each representing one of the two major genogroups of human noroviruses [5]–[7]. The receptor-binding interfaces of the two strains differ significantly in their structures, precise locations, receptor-binding modes, and amino acid compositions, although both locate on the top of the arch-like P dimer of the viral capsids [8]. However, sequence alignment showed that the key residues responsible for HBGA binding are highly conserved among strains within but not between the two genogroups, while the remaining sequences of the P2 subdomain are highly variable [8] (Figure 1). These data indicate that HBGAs play an important role in norovirus evolution, although other factors, such as host immunity, may also be involved. Each of the two genogroups represents an evolutionary lineage characterized by distinct genetic traits. Strains within each lineage have further diverged into sub-lineages (genotypes), probably by functional selection or adaptation through structural constraints of the human HBGAs. The polymorphic human HBGAs are most likely the driving force of the divergence of human noroviruses.

## Recognition of Carbohydrate Receptors May Be a Common Feature of Caliciviruses

The initial study of a calicivirus receptor was performed on an animal calicivirus, the rabbit hemorrhagic disease virus (RHDV) in genus *Lagovirus*, which recognizes the H-type 2 HBGA [9]. Field surveillance and epidemiology studies showed that this recognition is specific and associated with the resistance or susceptibility of rabbits with or without the H-type 2 antigen to the viruses [10]. Following the findings of the HBGA receptors for human noroviruses, several other caliciviruses have also been demonstrated to recognize a carbohydrate receptor. In genus *Norovirus*, the bovine norovirus (GIII) was recently shown to interact with HBGAs [11], while the murine norovirus (MNV, GV) recognizes the sialic acid [12]. In addition, the feline calicivirus (FCV) in genus *Vesivirus* uses the sialic acid on the host cell surface as a receptor, most likely for attachment [13]. Another receptor or co-receptor on the host cellular membrane, the junctional adhesion molecule-1 (JAM-1), was found to be required in FCV infection, probably helping virion penetration into host cells following the initial attachment [14]. Furthermore, the newly discovered rhesus monkey calicivirus, the Tulane virus, that was isolated from monkey stools [15], bound to human HBGAs [16].

Although further evidence for other genera of Caliciviridae, such as *Sapovirus,* is needed, the available data strongly suggest that the recognition of a carbohydrate receptor may be a common feature of caliciviruses, even though they have adapted to different host species after a long course of evolution. Increasing amounts of data also showed that many bacterial and other viral pathogens rely on a carbohydrate receptor for infection [17]. Thus, the requirement of a carbohydrate receptor could be a convergent factor in the evolution of these bacterial and viral pathogens. This principle is important not only for the research of human noroviruses that cause acute gastroenteritis, but also for other caliciviruses and other bacterial and viral pathogens that recognize similar carbohydrate receptors.

## Insight into the Epidemiology and Disease Control and Prevention of Norovirus Gastroenteritis

The findings of HBGA receptors as determinants of host range and evolution of noroviruses help our understanding of the epidemiology of norovirus gastroenteritis. The GII.4 (genogroup II, genotype 4) viruses have been found to predominant everywhere in the world in the past decade. Accordingly, in vitro binding assays revealed that most GII.4 strains recognized saliva of all ABO secretors that represent ∼80% of the general population. This could be an important reason for the predominance of this genotype over others that have narrower target populations. As a result of a long period of evolution, most strains in a genotype may have adapted to one or a few common epitopes of HBGAs. Thus, the consensus receptor-binding profiles of individual genotypes may not easily change. For example, a recent study showed that the major receptor-binding property of the GII.4 viruses to H-related antigens of secretors was traced back to a strain isolated as early as 36 years ago [18]. Our recent study also showed that all major genetic clusters of GII.4 viruses isolated in the current decade retained the consensus binding to H-related antigens [19], although changes in the HBGAs' binding profiles among GII.4 noroviruses have also been reported [20]. Such changes might offer the viruses new target populations, allowing the viruses to escape from host immunity. However, the significance of these variants in epidemiology remains to be determined. A critical question would be whether such variations become stable genetic traits that replace the currently dominant strains.

The possible role of herd immunity in norovirus evolution is another important issue for epidemiology. The surface region of the P2 domain around the highly conserved HBGA-binding interfaces changes significantly compared with other regions of the capsid and other viral proteins, suggesting a potential selection pressure from the host, such as acquired immunity. Emergence of new dominant GII.4 variants every 2–3 years that replace the previous ones [20], [21] also suggests antigenic changes of major circulating GII.4 strains over time. However, it is too early to conclude whether such variants represent antigenic shift or result in the emergence of new serotypes, as in the case of influenza viruses. Noroviruses clearly are not spread as rapidly and profoundly as influenza viruses because of less efficient transmission through the fecal/oral pathway compared with the respiratory pathway of flu. Noroviruses also may not induce a long-term immunity to build up persistent herd immunity as quickly as flu. Our understanding on GII.4 epidemiology and evolution is still in the initial stages and continual studies are necessary. It is an important issue because, if the epidemic variants represent only minor antigenic change (drifting), the vaccine strategy of an annual selection for flu vaccine may not be followed by a future norovirus vaccine.

The findings of the conservation of the HBGA-binding interfaces within the two major genogroups of human noroviruses are significant for the rational design and development of antivirals against these viruses. For example, a single compound that inhibits the function of the highly conserved HBGA-binding pocket may be capable of blocking infection of all strains that share the same or similar receptor-binding interfaces. Thus, only a few compounds might be sufficient to prevent infection of most human noroviruses causing acute gastroenteritis. Furthermore, a compound that is useful for the treatment of norovirus disease might also be effective for other bacterial and viral pathogens that recognize the same HBGA receptors.

## Issues with Animal Models in Norovirus Research

Caliciviruses are known for their genetic diversity with wide host ranges and tissue tropism, but many of them share common carbohydrate/HBGA receptors. The role of the HBGA receptor in viral evolution further raises the alert of zoonotic transmission of noroviruses, because many species share common HBGA receptors. In addition, noroviruses are highly adaptive due to a single-stranded RNA genome, high potency of genomic recombination, and the possible quasi-species nature of the genome. Furthermore, members of genus *Norovirus* that are able to infect animals have been identified, including the bovine, murine, and porcine noroviruses. Three genetic clusters of the porcine noroviruses have been classified in genogroup II of human noroviruses [22]. Finally, an animal reservoir of human noroviruses has been found in oyster and other bivalve shellfishes. Thus, further study on the origin and evolution of noroviruses and other caliciviruses is necessary for further understanding the virus–host interaction and potential risk of cross-species transmission of noroviruses, which is important for disease control and prevention.

Great efforts have been made in developing an animal model for human noroviruses. Several non-human primate species have been challenged with human noroviruses, such as rhesus macaque, pigtail macaque, and chimpanzee. Limited success has been observed for clinical infection and illness in non-human primates compared with the human host. These models are worth further evaluation owing to their genetic and phenotypic relatedness in many aspects with humans.

Gnotobiotic (Gn) pig is a more promising model of human noroviruses, and currently is under investigation and development. Pigs share several characteristics with humans in their gastrointestinal anatomy, physiology, immune system, and the presence of HBGAs, such as the A and H antigens on mucosal surfaces. In a neonatal Gn pig model, human norovirus infection has resulted in diarrhea, virus shedding, seroconversion, immuno-cytopathic change in the intestinal sections, and transient viremia [23]–[25]. Similar results have also been observed in Gn calves [26], suggesting that these Gn animal models may be useful for the study of immunology and pathogenesis and the assessment of vaccines and antivirals against human noroviruses.

The murine norovirus (GV) [27] has been used as a surrogate to study the pathogenesis, immunology, and replication of human noroviruses, and a great amount of data have been generated. However, the limitations of this model are obvious due to the difference between the two viruses in clinical manifestations (without diarrhea/vomiting), host receptors (sialic acid versus HBGAs), infected cell types (dendritic/macrophages versus digestive epithelial cells), and pathogenesis. Thus, an ultimate understanding of human noroviruses and assessment of intervention approaches will most likely rely on the establishment of an effective animal model of human noroviruses. A further animal surrogate model may be a rhesus monkey calicivirus, the Tulane virus. This enteric virus can replicate in vitro in monkey cell lines [15]. Most importantly, the Tulane virus recognizes human HBGAs [16]. A weakness of this model is that the Tulane virus belongs to a unique genus separate from the *Norovirus* genus, and it remains unknown whether the Tulane virus causes gastroenteritis like human noroviruses.

## Additional Questions on the Host Interaction of Noroviruses

As a potential key factor in co-evolution between many microorganisms and human hosts, the polymorphic human HBGA system may be the result of selection by some highly virulent or life-threatening bacterial or viral pathogens in the past. Noroviruses do not belong to these pathogens because currently they lead only to the modest disease of acute gastroenteritis. However, this cannot exclude the possibility that noroviruses were once highly virulent in the past and/or may become so in the future, because noroviruses are among those highly adaptive species. The emergence of the highly virulent RHDV that almost eradicated entire rabbit colonies in China and European countries in the 1980s is a good example. The epidemic of SARS in 2003 could be another warning.

Noroviruses are still difficult to cultivate in vitro, even after the discovery of HBGA receptors. One possibility is that a functional co-receptor necessary for norovirus replication is missing in the cell culture, although failures of additional downstream steps of viral replication also may be the reason. In FCV, both sialic acid and JAM-1 are required for viral replication, in which sialic acid is believed to be a ligand or receptor for virion attachment, while the membrane protein JAM-1 may function as a co-receptor to facilitate FCV penetrating into the host cells. Since this two-step process has also been shown in other viruses such as the reovirus [28], and a membrane protein has been demonstrated to interact with human noroviruses, it would be significant to explore the two-step process to search for and characterize such a co-receptor for noroviruses.

**Figure 2:**
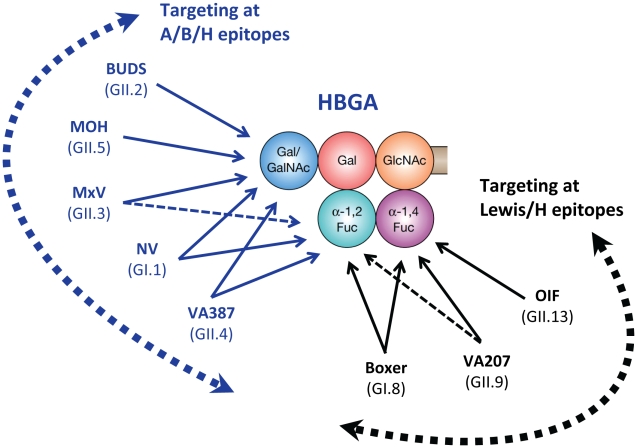
Schematic interactions and relationships among different human noroviruses with a complete product of human HBGA. Representative strains of different genotypes in the two major genogroups (GI and GII) of human noroviruses are shown according to their target saccharides. Arrows indicate interactions between individual noroviruses and specific residues of human HBGAs. Dashed lines indicate a weaker interaction. The five circles in different colors represent the five saccharide residues of a complete product of an H-related HBGA (H, A, B, Le^b^, or Le^y^). The curved dashed arrows indicate two major binding groups, the A/B/H (blue) and the Lewis/H (black) binding groups, according to their target residues on human HBGAs. The binding specificity and affinity of these norovirus strains were determined in [30].

The role of norovirus VP1 in interaction with host receptors has been well studied. Little is known, however, about VP2, the minor structural protein of the capsid. The fact that VP2 has a similar or higher variation compared to VP1 suggests that it might also involve a norovirus–host interaction. In addition, increasing amounts of data showed that genomic recombination occurs frequently among human noroviruses, with a breakpoint mainly between the non-structural and structural genes. This would confer recombinant variants with new genetic traits with possible survival advantages. Finally, although human noroviruses are highly diverse in recognition of HBGAs, only minor structural differences in their HBGA-binding interface with shared HBGA epitopes are expected among genetically closely related strains (Figure 2). For example, the GII.3 viruses, such as strain MxV, share common bindings to type A and B saliva, with only slightly weaker binding affinities to saliva of type O secretor compared with the consensus H binding (A, B, and O secretors) of GII.4 viruses. GII.3 has been found to predominate second only to GII.4 viruses in many countries, and GII.3 appeared to be the most predominant genotype in the 1970s [18]. In the laboratory a single residue mutation around receptor-binding interfaces can result in a change of HBGA binding patterns [8], [29]. Thus, it would be of significance to explore whether the consensus receptor binding patterns can switch between two genotypes in nature and whether GII.4 noroviruses will continue to dominate or will be replaced by other genotypes in future epidemics.
